# Role of Vitamin D Deficiency in Increased Susceptibility to Respiratory Infections Among Children: A Systematic Review

**DOI:** 10.7759/cureus.29205

**Published:** 2022-09-15

**Authors:** Anjumol Raju, Gaurav Luthra, Mahrukh Shahbaz, Halah Almatooq, Paul Foucambert, Faith D Esbrand, Sana Zafar, Venkatesh Panthangi, Adrienne R Cyril Kurupp, Safeera Khan

**Affiliations:** 1 Pediatrics, California Institute of Behavioral Neurosciences & Psychology, Fairfield, USA; 2 Internal Medicine, California Institute of Behavioral Neurosciences & Psychology, Fairfield, USA; 3 Dermatology, California Institute of Behavioral Neurosciences & Psychology, Fairfield, USA

**Keywords:** rickets, immunomodulatory effect of vitamin d, children, respiratory infections, vitamin d deficiency

## Abstract

Vitamin D has several roles in the immune system besides its effects on bone metabolism. Acute respiratory infections are common infections in children. Severe lower respiratory tract infections (LRTIs) even cause death in children, especially in those less than five years of age. Our study aims to examine whether children with vitamin D deficiency are susceptible to respiratory infections and to study the association between vitamin D deficiency and the severity of respiratory infections. We comprehensively searched research articles in PubMed, ScienceDirect, and Cochrane library databases. The main keywords were vitamin D deficiency, respiratory infections, and children. We used Preferred Reporting Items for Systematic Reviews and Meta-Analyses (PRISMA) 2020 guidelines to conduct this systematic review. The initial search showed 16,120 papers. A meticulous screening of research articles using the eligibility criteria and quality appraisal tools was done. Finally, 10 research articles qualified for this systematic review, including eight case-control studies, one randomized controlled trial (RCT), and one cohort study. Seven of 10 research studies reviewed found that children with low vitamin D levels are susceptible to respiratory infections. Five studies discussed the severity of respiratory infections and low vitamin D levels. This systematic review concluded that children with low vitamin D levels are prone to developing respiratory infections. But we could not find a conclusive association between the severity of respiratory infections and low vitamin D levels.

## Introduction and background

Vitamin D deficiency has emerged as a pandemic in the modern world [1]. According to recent studies, over a billion people all around the globe are suffering from vitamin D deficiency [2]. Vitamin D is a steroidal hormone that plays a vital role in bone metabolism [3]. New clinical studies describe the modulatory effects of vitamin D on the innate immune system and defensive function against many infections like pneumonia [4].

Upper respiratory tract infections (URTIs) can be non-serious but can evolve as an epidemic in communities. Lower respiratory tract infections (LRTIs) can progress into severe infections and are recognized as one of the primary causes of mortality in children under five years of age. Viruses are the principal pathogen in children's URTIs and LRTIs [5].

The respiratory tract epithelium is prone to invasions of pathogens because of its vast surface area. The primary cells involved in the defense mechanism in the respiratory system include the airway epithelia, alveolar macrophages, and dendritic cells. All these cells contain the gene cytochrome P450 family 27 subfamily B member 1 (CYP27B1), which helps express the vitamin D receptors on the cell surface and produces the 1-alpha hydroxylase enzyme. This enzyme converts vitamin D into the active form 1, 25-dihydroxy vitamin D (1, 25 (OH)_2_ vitamin D) [6]. The 1, 25 (OH)_2 _vitamin D produced in the airway epithelial cells induces cell proliferation and inhibition of apoptosis following an inflammatory episode. Loss of vitamin D receptors from the surface of these cells causes damage to the integrity of the epithelium [6]. Vitamin D also induces the gene expression of Toll-like receptors (TLRs). They are the primary responders who recognize the components of bacterial cell walls and genome [7].

Cathelicidin and beta-defensin 2 are two important antimicrobial peptides in neutrophils, monocytes, natural killer cells, and respiratory tract epithelial cells lining. Vitamin D stimulates the expression of these antimicrobial peptides [8]. LRTIs are prevalent during the winter season, pointing to the impact of weather on the development of these diseases. The ultraviolet radiation exposure decreases during the winter, reducing vitamin D levels in the body. This may lead to a higher incidence of LRTIs during the winter season [9].

The postulate of vitamin D deficiency as a risk factor for developing respiratory infections is supported by the high incidence of pneumonia in children with rickets in a study conducted in Ethiopia [10]. However, another study showed that vitamin D supplementation has no impact on preventing tuberculosis or acute respiratory infections in children [11]. So there are mixed opinions about the role of vitamin D and respiratory infections in children [3]. This study aims to find if vitamin D deficiency is a risk factor for developing respiratory infections in children and investigate the association between low vitamin D levels and the severity of respiratory infections.

## Review

Methods

Preferred Reporting Items for Systematic Reviews and Meta-Analyses (PRISMA) 2020 guidelines were used to conduct this systematic review [12]. The protocol is described as follows.

Eligibility Criteria

We included research articles about the pediatric population (newborn period to 18 years of age), full-text articles written in English, and articles relevant to the topic. Research conducted on the adult population and articles in other languages were excluded.

Search Strategy

A systematic search on PubMed, Cochrane library, and ScienceDirect yielded sufficient research papers on our topic of interest. We used Medical Subject Headings (Mesh) keywords in PubMed and advanced search in Cochrane library and ScienceDirect to extract the relevant research articles on vitamin D deficiency and respiratory infections in children. The following search keywords were used in each database: a) PubMed: ("Vitamin D Deficiency/complications"[Mesh] OR "Vitamin D Deficiency/physiopathology"[Mesh]) AND ("Respiratory Tract Infections/etiology"[Mesh] OR "Respiratory Tract Infections/pathology"[Mesh]); b) Cochrane library: ("vitamin D deficiency") AND (respiratory infections) AND ("Child"); and c) ScienceDirect: Vitamin D deficiency AND respiratory infections AND Children.

**Figure 1:**
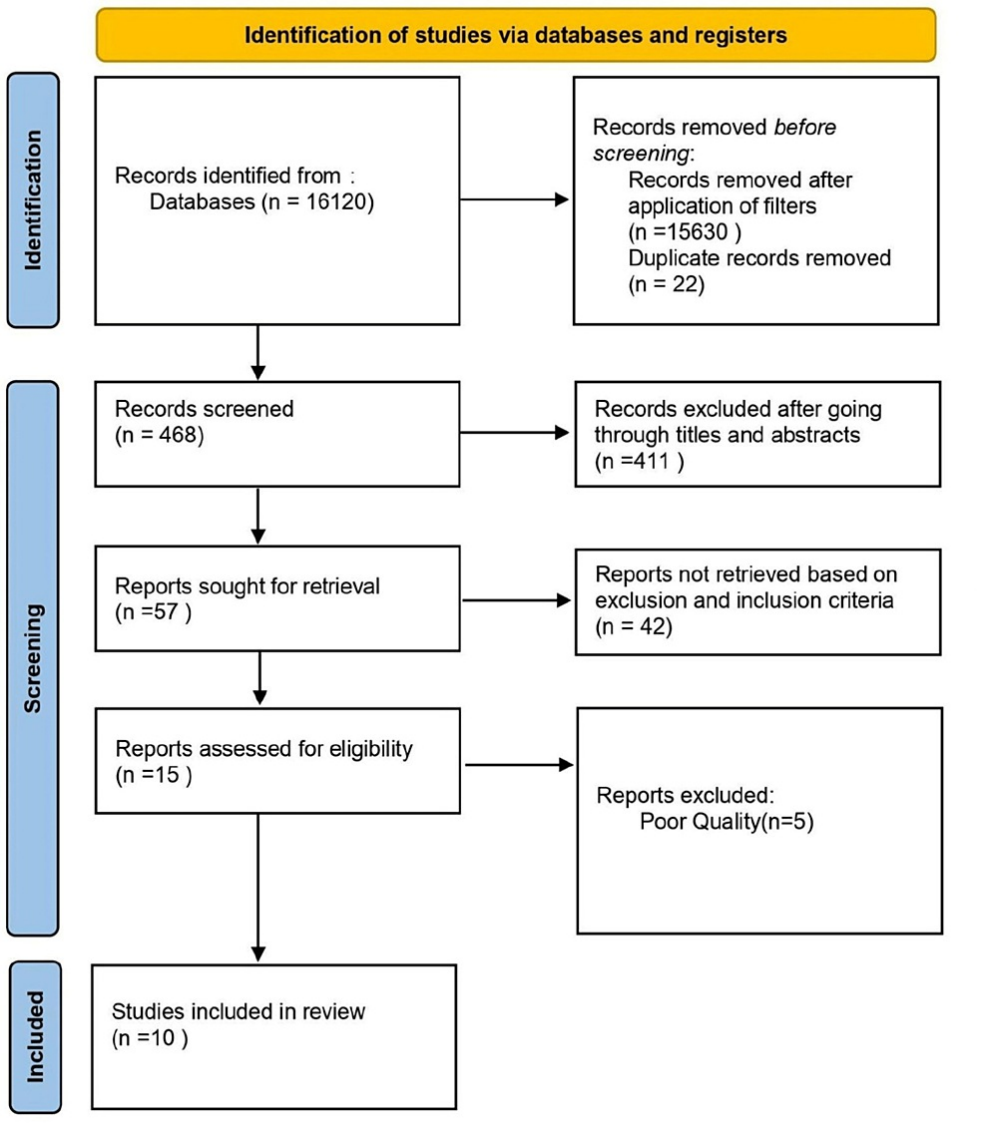
Flowchart shows the process involved in selecting the research articles n: number of articles.

The initial search yielded 16,120 research articles, and we applied the following filters in PubMed and ScienceDirect to find the relevant research articles: PubMed: species, humans; language: English; and age: childbirth to 18 years; and ScienceDirect: publication title: journal pediatrics, and article type: research and review articles. The last search on all research papers was on April 15, 2022. The PRISMA flowchart (Figure 1 ) shows the process of selecting research articles for this systematic review.

Results

The initial search on all three databases showed 16,120 papers. After applying appropriate filters and removing duplicates, 468 research papers were selected. AR and GL conducted screening of the research articles independently in two phases. The first phase of screening was done by reading the titles and abstracts. During the second screening phase, researchers went through 57 research papers and excluded 42 articles that did not meet the eligibility criteria. A consensus was achieved among the two authors while selecting the articles. In the end, there were 15 studies, which qualified for final quality checking. These were eight case-control studies, one cohort study, one case series study, two cross-sectional studies, one randomized controlled trial (RCT), one systematic review, and one literature review.

Quality Assessment

We used respective Joanna Briggs Institute (JBI) quality appraisal tools for RCT, case-control, cross-sectional, case series, and cohort studies. The Assessment of Multiple Systematic Reviews 2 (AMSTAR-2) checklist was used for systematic reviews, and the Scale for the Assessment of Narrative Review Articles (SANRA) checklist was used for literature reviews. The accepted score for qualifying a study article to include in our systematic review was set as ≥70%. A total of 10 studies were identified as good-quality studies for our systematic review.

Description of the Included Studies 

The total number of patients included in this systematic analysis is 11,565. The final research articles include eight case-control studies [3,9,10,13-17], one RCT [11], and one cohort study [18]. The age of the study participants ranged from newborn period to 16 years of age. Most of the studies reported the serum vitamin D status of the study participants [3,9,11,13-15]. Two studies described vitamin D deficiency as clinical and radiological evidence of rickets [10,16]. Tables 1-3 show the characteristics of the included studies.

**Table 1:** The characteristics of the case-control studies included in this systematic review COVID-19: coronavirus disease 2019, LRI: lower respiratory infection, ICU: intensive care unit, ED: emergency department, CAP: community-acquired pneumonia, SD: standard deviation, N/A: not applicable.

Author and year	Alpcan et al. 2021 [13]	Dinlen et al. 2016 [3]	El-Radhi et al. 1982 [16]	Golan-Tripto et al. 2021 [14]	Li et al. 2018 [15]	McNally et al. 2009 [9]	Muhe et al. 1997 [10]	Roth et al. 2010 [17]
Study setting	COVID-19 patients vs healthy children.	Term newborns with LRI admitted in ICU vs healthy newborns.	Patient admitted with wheezy bronchitis vs children from the vaccination clinic.	Children visited ED with acute bronchiolitis vs children with non-respiratory febrile illness.	Children with CAP vs healthy children.	Children admitted with bronchiolitis or pneumonia vs children receiving care at ambulatory, ED, and in-patient units.	Children admitted to the hospital due to pneumonia vs children admitted with no pneumonia.	Children admitted to the ward with respiratory illness vs children without respiratory illness.
Period of study	May 2020- to December 2020	October 2013 to March 2014	N/A	N/A	January 2009 to December 2011	November 2007 to May 2008	January 1989 to December 1993	January 2008 to February 2008
Sample size cases/control	75/80	30/30	100/100	80/47	797/785	105/92	500/500	25/25
The age range of study participants (cases vs controls)	10.7 ± 5.5 vs 9.9 ± 4.6 years	12.2 ± 4.6 vs 10.3 ± 5.2 days	3-12 months	5 (3-9) vs 9 (5-16) months	3.15 (0.01-14.0) vs 3.28 (0.1-12.0) years	13.8 ± 15.2 vs 13.4 ± 13.8 months	13.6 (SD: 8.3) vs 13.4 (SD: 8.5) months	1-18 months
Vitamin D status (cases vs controls)	21.5 ± 10 vs 28 ± 11 IU	9.5 (7.9-12.2) vs 15.5 (12-18) ng/mL	N/A	28 (18-52) vs 50 (25-79) nmol/L	19.04 ± 9.86 vs 31.71 ± 14.82 ng/mL	81 ± 39 vs 83 ± 30 nmol/L	N/A	29.1 (SD: 17.2) vs 39.1 (SD: 9.4) nmol/L
Vitamin D deficiency (cases vs controls)	44% vs 17.5%	86% vs 56%	N/A	73% vs 51%	56% vs 20%	24% vs 16%	N/A	N/A
Vitamin D insufficiency (cases vs controls)	40% vs 48.8%	N/A	N/A	16% vs 21%	28% vs 31%	44% vs 35%	N/A	N/A
Vitamin D sufficiency (cases vs controls)	16% vs 33.7%	N/A	N/A	11% vs 28%	16% vs 49%	32% vs 49%	N/A	N/A
Study outcomes	The levels of vitamin D were lower among cases.	The median vitamin D levels were lower in the study group than in the control group.	The children with wheezy bronchitis group had two and a half times of incidence of rickets than the control group.	The vitamin D levels were lower in the bronchiolitis group than in the control group.	The vitamin D levels are lower in the CAP group.	The vitamin D levels were similar in both groups.	The incidence of rickets in children with pneumonia is 13 times more compared to the control group.	The vitamin D levels are lower in some cases.

**Table 2:** The characteristics of the cohort study included in this systematic review CAP: community-acquired pneumonia.

Author and year	Huang et al. 2017 [18]
Study setting	Retrospectively analyzed data of children admitted with CAP
Period of study	October 2011 to September 2012
Sample size total/cohort of vitamin D deficient patients/cohort of patients without deficiency	77/55/22
Age of study participants	3.5 years (0.3-12) median age
Study outcome	Vitamin D status in CAP children is inversely related to age

**Table 3:** The characteristics of RCT included in this systematic review QFT: QuantiFERON in tube assay, TB: tuberculosis, ARI: acute respiratory infection, RCT: randomized controlled trial.

Author and year	Ganmaa et al. 2020 [11]
Study setting	QuantiFERON in tube assay (QFT)-negative children were randomly assigned to the intervention (vitamin D) group and placebo group
Period of study	September 2015 to March 2017
Sample size total/intervention/placebo	8,851/4,418/4,433
Age of study participants	9.4 ± 1.6 years
Vitamin D status at the beginning of the trial	11.9 ± 4.2 ng/mL
Vitamin D status at the end of the trial: intervention/placebo	31 ± 91/10.7 ± 5.3 ng/mL
Participants who tested positive for QFT assay at the end of the trial: intervention/placebo	3.6%/3.3%
Participants who reported ≥1 episode of ARI: intervention/placebo	86%/85.9%
Outcome	Vitamin D supplementation does not have any effect on preventing TB infections and ARIs

Discussion

Vitamin D and the Immune System

**Figure 2:**
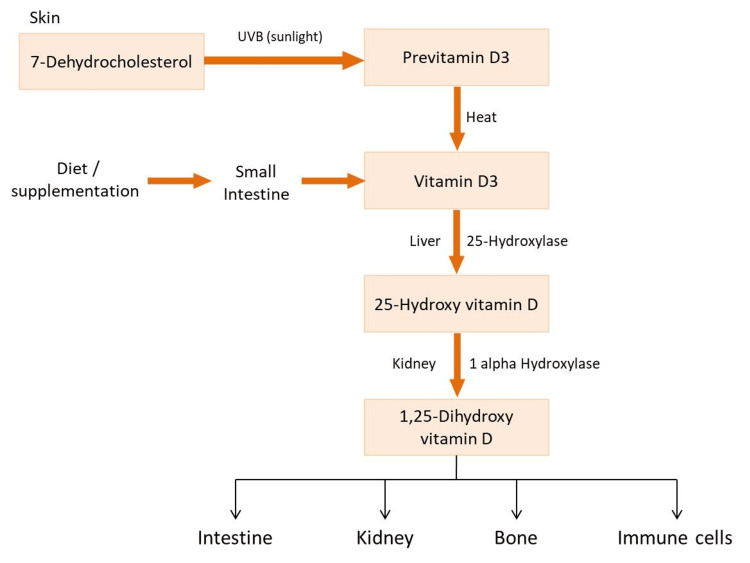
The synthesis and metabolism of vitamin D UVB: ultraviolet B. Figure created by Anjumol Raju.

Vitamin D is a fat-soluble vitamin. The human body produces 90% of vitamin D through exposure to sunlight. The rest of the 5% of the body's vitamin D supply depends on our diet, specifically food products such as dairy products and dark fish. The sun's ultraviolet B (UVB) radiation converts 7-dehydrocholesterol to pre-vitamin D3, forming vitamin D3 [19]. The liver enzyme cytochrome P-450 then converts vitamin D3 into 25-hydroxyvitamin D (25-OH vitamin D) [13]. 25-Hydroxyvitamin D then undergoes hydroxylation in the kidney by one alpha-hydroxylase enzyme to form the 1, 25-dihydroxy vitamin D (1, 25 (OH)_2_ vitamin D) or calcitriol, which is the biologically active form. The active form of vitamin D then acts on vitamin D receptor (VDR), which is expressed not only in bone and intestine but also in bone marrow, brain, pancreas, prostate, tumor cells, and immune cells. This concludes that vitamin D has a wider spectrum of functions not limited to maintaining calcium homeostasis [20]. The synthesis and metabolism of vitamin D are discussed in Figure 2 [1,19].

Vitamin D modulates various aspects of innate immunity. It is involved in expressing pattern recognition receptors (PRRs), also called Toll-like receptors (TLRs). PRRs are the initial responders that identify the structures called pathogen-associated molecular patterns (PAMPs). Various components of the bacterial cell wall, cell membrane, and genome constitute PAMPs [7,21]. TLRs present on immune system cells such as natural killer cells, mast cells, macrophages, eosinophils, neutrophils, dendritic cells, and basophils. The stimulation of TLRs by PAMPs activates signaling pathways by the host against the pathogen and restores the injured tissue. The activation of Toll-like receptor (TLR) cascades the release of numerous inflammatory cytokines and immune modulators such as interleukin-1β and interleukin-8, a neutrophil chemokine. These reactions help cells of innate immunity to convey the message about invading organisms to other parts of the immune system [7,22].

Vitamin D also activates the gene encoding cluster of differentiation 14 (CD14), a cofactor of the PRR Toll-like receptor-4 (TLR4) [7]. Lastly, vitamin D-vitamin D receptor (VDR) binding increases the gene expression of antimicrobial peptides (AMPs), which include cathelicidin and beta-defensin-2 [7,23]. All these mechanisms indicate that vitamin D signaling activates the expression of various steps of a strong immune response.

Vitamin D Deficiency and Respiratory Infections

Most of the research papers in our systematic review define vitamin D deficiency as patients with 25-OH vitamin D levels less than 20 ng/mL (<50 nmol/L) [9,13-15]. Those with 25-OH vitamin D levels of 21-30 ng/mL (52.5-75 nmol/L) were categorized under vitamin D insufficiency [9,14,15], and those having 25-OH vitamin D levels of more than 30 ng/mL (>75 nmol/L) were categorized under adequate vitamin D level [9,13-15]. Two studies described rickets as vitamin D deficiency [10,16], while one defined vitamin D deficiency as 25-OH vitamin D levels below 15 ng/mL [3].

There are two recent case-control studies included in our systematic review. Alpcan et al. studied vitamin D deficiency in coronavirus disease 2019 (COVID-19) patients and healthy controls in 2020 in Turkey [13]. They found that the incidence of vitamin D deficiency was more common in the study group (44%) vs the control group (17.5%). Also, the average serum concentrations of vitamin D were lower in COVID-19 patients than in healthy controls [13]. Golan-Tripto et al. conducted the second study in Israel (2020) [14]. The study showed lower vitamin D levels in the bronchiolitis group (cases) than in the non-respiratory febrile illness group (controls) [14]. Roth et al. also demonstrated the same results in a study conducted on children of rural Bangladesh [17]. They found that the patients with acute lower respiratory infection had significantly lower serum 25-OH vitamin D than the control group [17].

Dinlen et al. studied the association between acute lower respiratory infections and vitamin D deficiency in newborns (2016) [3]. The study concluded that the lower blood 25-OH vitamin D level might increase a newborn's risk of acute respiratory infections. The study also describes a significant association between maternal and neonatal vitamin D levels, hence suggesting an adequate diet, vitamin D supplementation, and getting enough sunlight for pregnant women [3]. A case-control study by Li et al. on children with pneumonia also substantiates the abovementioned results [15]. There was a lower serum 25-OH vitamin D among pneumonia patients than in the control group [15]. Another large study conducted on Ethiopian children about the risk of developing pneumonia and nutritional rickets revealed that children with pneumonia had 13 times increased incidence of rickets than the corresponding control group [10].

A similar study from Iraq examined the association between rickets and bronchitis among infants. They found that the incidence of radiologically proven rickets was seen more in children with wheezy bronchitis than in children in the control group. The breastfeeding practices among the bronchitis group explain the high incidence of rickets in that group [16]. In contrast to all of the studies mentioned above, McNally et al. found no statistical difference in the level of vitamin D among children with acute lower respiratory infections and children with non-respiratory illnesses [9].

A three-year randomized controlled trial was conducted by Ganmaa et al. on school children of Mongolia about the effect of vitamin D supplementation and the risk of the development of tuberculosis or acute respiratory infection [11]. The study revealed that the intervention of 14,000 international unit (IU) weekly oral dose of vitamin D has no effect in reducing the risk of development of tuberculosis or acute respiratory infection [11]. Most of the research articles we reviewed in our systematic review concluded that children with low vitamin D levels are more prone to acute respiratory infections.

Vitamin D Deficiency and Disease Severity

Alpcan et al. observed that the COVID-19 patients with a low vitamin D level had developed fever and cough at a significantly lower rate [13]. This observation attributes that a sufficient vitamin D level is crucial for showing symptoms such as fever and cough, speculating the role of vitamin D levels in inflammatory and immune reactions. The study also observed a positive correlation between vitamin D levels and factors like white blood cell (WBC) count, lymphocyte count, and platelet count and a negative correlation with the duration of hospital stay [13].

Golan-Tripto et al. studied vitamin D deficiency and bronchiolitis in infants and toddlers [14]. The investigators used a Modified Tal Score (MTS) to assess the severity of bronchiolitis. They could not observe an association between vitamin D and the intensity of bronchiolitis or length of hospital stay. But one interesting finding in this study is that two patients with severe bronchiolitis (those with the MTS ≥ 11) or patients with an extended hospital stay had insufficient 25-OH vitamin D levels. So the authors concluded that vitamin D could be considered an indicator of disease severity [14].

In contrast to the study mentioned above, Li et al. found that vitamin D deficiency is a clear indicator of disease severity [15]. This study infers that the patients with sepsis are relatively more deficient/severely deficient in vitamin D than the patients with pneumonia. They also demonstrated that children with low vitamin D levels might be vulnerable to mechanical ventilation and multiple organ dysfunction syndrome (MODS) [15]. These study findings are relevant because of the relatively large sample size compared to other studies.

Another study conducted by McNally et al. demonstrated that patients with acute lower respiratory infections (ALRIs) who needed intensive care unit admission had significantly lower levels of vitamin D than the patients admitted to general wards [9]. But this study could not find a statistically significant association between vitamin D deficiency and ALRIs. This implies that vitamin D has no role in the development of mild-moderate acute lower respiratory infection, but it has a definite role in the progression of the disease [9]. A case-control study from Ethiopia showed no significant difference in mortality between children with pneumonia and rickets and those without rickets. They also found that cases were more malnourished when compared to the control group, and the most frequent category of malnourishment was marasmus [10]. Since the research articles showed mixed opinions about vitamin D deficiency and disease severity, we could not infer a conclusion regarding the relationship between vitamin D deficiency and the severity of respiratory infections.

Trends of Vitamin D Status and Age

There is an inverse relationship between vitamin D levels and age among children. The study conducted in Turkey among COVID-19 subjects showed that children with vitamin D deficiency were older than children with adequate vitamin D levels. Also, this study found a negative correlation between age and vitamin D status [13]. Another study by Huang et al. also found the same trend of decreasing serum 25-OH vitamin D levels with increasing age of children [18]. Li et al. also demonstrated the negative association between vitamin D status and age in children [15]. Nowadays, older children tend to stay indoors due to the heavy academic curriculum and the increased popularity of indoor games and electronic devices.

Limitations

The age group of children included in this systematic review ranges from the newborn period to adolescents. This heterogeneity of age may affect the outcomes of each study since the immune system is still developing in newborns and is well-developed in the older age group. Two of the studies described rickets as an indicator of vitamin D deficiency. The rest of the studies in this systematic review assessed their study participants' serum 25-OH vitamin levels. This difference in the measure of vitamin D levels can lead to a measurement bias.

## Conclusions

Our systematic review aims to study the role of vitamin D deficiency as a risk factor for respiratory infections in children. Also, we tried to explore the association between vitamin D deficiency and the severity of respiratory infections. Ten research articles were selected and analyzed for this systematic review. Our study has shown that children with vitamin D deficiency are more prone to respiratory infections, and the prevalence of vitamin D deficiency increases with age. However, we could not find a conclusive association between vitamin D deficiency and the severity of the infections. Therefore, it is essential to monitor the vitamin D levels annually in children and supplement vitamin D in children with deficiency. Parents and children should be educated about the importance of adequate sunlight exposure and including vitamin D-rich foods in their diet. If taking vitamin D supplements, an optimal dose should be maintained to prevent potential vitamin D toxicity.
